# Cross-Linked Hyaluronate and Corticosteroid Combination Ameliorate the Rat Experimental Tendinopathy through Anti-Senescent and -Apoptotic Effects

**DOI:** 10.3390/ijms23179760

**Published:** 2022-08-28

**Authors:** Po-Yen Ko, Che-Chia Hsu, Shih-Yao Chen, Li-Chieh Kuo, Wei-Ren Su, I-Ming Jou, Fong-Chin Su, Po-Ting Wu

**Affiliations:** 1 Department of Orthopedics, National Cheng Kung University Hospital, College of Medicine, National Cheng Kung University, Tainan 70428, Taiwan; 2 Department of Nursing, College of Nursing, Chung Hwa University of Medical Technology, Tainan 717302, Taiwan; 3 Department of Occupational Therapy, National Cheng Kung University, Tainan 701401, Taiwan; 4 Medical Device Innovation Center, National Cheng Kung University, Tainan 701401, Taiwan; 5 Department of Orthopedics, College of Medicine, National Cheng Kung University, Tainan 701401, Taiwan; 6 Department of Orthopedics, E-Da Hospital, Kaohsiung 82445, Taiwan; 7 School of Medicine, College of Medicine, I-Shou University, Kaohsiung 82445, Taiwan; 8 GEG Orthopedic Clinic, Tainan 74543, Taiwan; 9 Department of Biomedical Engineering, National Cheng Kung University, Tainan 701401, Taiwan; 10 Department of Biochemistry and Molecular Biology, College of Medicine, National Cheng Kung University, Tainan 701401, Taiwan

**Keywords:** cross-linked hyaluronate, dexamethasone, senescence-associated secretory phenotype, inflammation, rat tendinopathy model

## Abstract

The combination of cross-linked hyaluronate (cHA) and corticosteroid showed more rapid pain or functional improvement in knee osteoarthritis and adhesive capsulitis. However, rare evidence of this combination in treating tendinopathy has been reported. We hypothesized that the specific formulations of cHA and dexamethasone (DEX) conferred amelioration of tendinopathy via anti-apoptosis and anti-senescence. In this controlled laboratory study, primary tenocytes from the human tendinopathic long head of biceps were treated with three cHA formulations (cHA:linealized HA = 80:20, 50:50, and 20:80) + DEX with or without IL-1β stimulation. Cell viability, inflammatory cytokines, tendon-related proliferation markers, matrix metalloproteinases (MMPs), senescent markers, and apoptosis were examined. The in vivo therapeutic effects of the selected cHA + DEX combinations were evaluated in a collagenase-induced rat patellar tendinopathy model. The expression levels of inflammatory mediators, including IL-1β, IL-6, COX-2, MMP-1, and MMP-3 were significantly reduced in all cHA + DEX-treated tenocytes (*p* < 0.05, all). The cHA (50:50) + DEX and cHA (20:80) + DEX combinations protected tenocytes from cytotoxicity, senescence, and apoptosis induced by DEX in either IL-1β stimulation or none. Furthermore, the two combinations significantly improved the rat experimental tendinopathy by reducing ultrasound feature scores and histological scores as well as the levels of apoptosis, senescence, and senescence-associated secretory phenotypes (*p* < 0.05, all). We identified two specific cHA formulations (cHA (50:50) and cHA (20:80)) + DEX that could ameliorate tendinopathy through anti-senescence and -apoptosis without cytotoxicity. This study provides a possible approach to treating tendinopathy using the combination of two well-known agents.

## 1. Introduction

Tendinopathy is a chronic musculoskeletal disorder claimed to account for 30–50% of all sports-related injuries [1]. However, the exact pathological mechanism remained unclear. It is widely accepted that inflammation and degeneration may mutually affect disease progression [2]. Furthermore, emerging evidence shows that excess apoptosis and senescence play important roles in the pathogenesis of tendinopathy [3,4,5,6]. Therefore, ameliorating apoptosis [7] and senescence [6,8] in the translation studies would be the targeted therapeutic approach for tendinopathy.

Conservative treatment, including physical therapy, injection and extracorporeal shock wave, is usually suggested as the first-line management [9]. Corticosteroid is one of the most common agents in local injection and showed its short-term therapeutic effects compared with other treatments [10,11,12]. However, reported cytotoxicity [13], increased senescence [14], decreases in extracellular matrix (ECM) synthesis [15] in vitro, collagen necrosis and disorganization, as well as a reduction in mechanical properties in vivo [15] will limit the clinical practice of corticosteroid.

Hyaluronic acid (HA) enhances the biological activities of fibroblasts, including ECM synthesis and cell proliferation [16], and has been reported to treat tendinopathy effectively in clinical practice [17,18,19] and animal models [20,21]. Recently, the cross-linking technique can slow the in vivo degradation of HA and facilitate the clinical treatment in a single injection [22]. Interestingly, the combination of cHA and corticosteroid showed more rapid pain or functional improvement in knee osteoarthritis (OA) [23,24,25], and more effective in improving functional scores at one month in adhesive capsulitis [26]. However, there is a paucity of evidence in the treatment of tendinopathy using the combination of cHA and corticosteroid. In response, we aimed to evaluate the safety and the possible therapeutic effects of the various cHA formulations combined with dexamethasone (DEX) in human tenocytes and a rat patellar tendinopathy model. We hypothesized that the specific formulation of cHA and DEX conferred amelioration of tendinopathy via anti-apoptosis and anti-senescence.

## 2. Results

### 2.1. Tenocyte Viability and Collagen Synthesis

**Figure 1:**
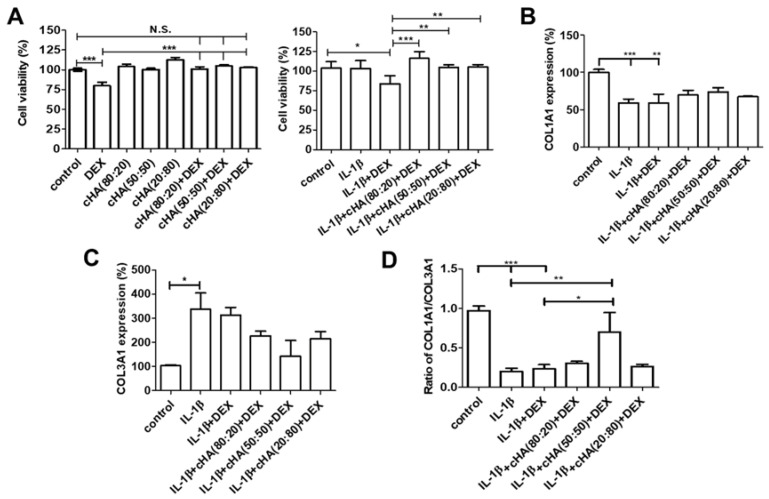
Effects of various combinations of cross-linked hyaluronate (cHA) with dexamethasone (DEX) in cell viability and interleukin-1 (IL)-1β-stimulated gene expression. Human primary tenocytes were treated with different formulations of cHA (cHA:linearized HA = 80:20, or 50:50, or 20:80, at the concentration of 2.5 mg/mL), DEX (100 μM), and combinations of cHA with DEX (100 μM) in response to IL-1β (1 ng/mL) stimulation. (**A**) Cell viability was determined by WST-8 analysis. Data were represented as the percentages normalized with the average WST-8 values read at an absorbance of 450 nm of the control group and expressed as mean ± SEM. Relative expression of (**B**) COL1A1 (**C**) COL3A1 was determined by quantitative reverse transcription polymerase chain reaction (qRT-PCR). (**D**) The ratio of COL1A1/COL3A1 was shown among each group. Values are the mean ± SEM (*n* = 3–8). * *p* < 0.05, ** *p* < 0.01, *** *p* < 0.001.

To examine the effects of the three cHA + DEX formulations (cHA (80:20) + DEX, cHA (50:50) + DEX, and cHA (20:80) + DEX) in human primary tenocytes, cells were treated with various cHA + DEX formulations or DEX alone in response to IL-1β stimulation or none. In contrast to significant cytotoxicity induced by DEX, neither of the cHA + DEX formulations induced cytotoxicity in either IL-1β stimulation or none (Figure 1A) after 5-day treatment. For collagen synthesis, treatment of either IL-1β or ILβ + DEX induced a significant decrease in COL1A1 expression and an increase in COL3A1 expression, and eventually decreased the ratio of COL1A1/COL3A1 in tenocytes. However, all three cHA + DEX formulations reversed the expression fashion of COL1A1 and COL3A1, but the effects did not reach statistical significance (Figure 1B,C). Only cHA (50:50) + DEX significantly increased the expression ratio of COL1A1/COL3A1 compared with either IL-1β or ILβ + DEX treatment (*p* < 0.01, Figure 1D).

### 2.2. Inflammatory Mediators and MMPs in Tenocytes

**Figure 2:**
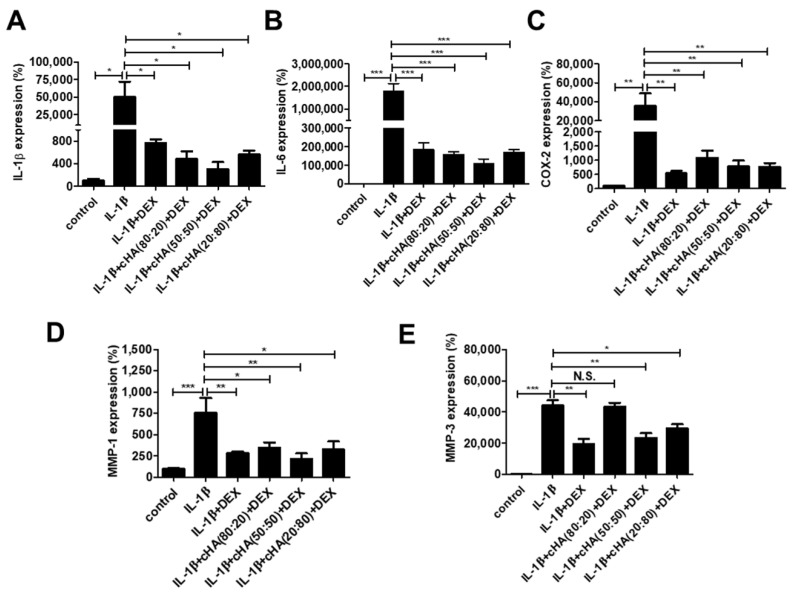
Effects of cHA with DEX in IL-1β-stimulated expressions of inflammatory mediators and matrix metalloproteinases (MMPs). Human primary tenocytes were treated with different formulations of cHA (cHA:linearized HA = 80:20, or 50:50, or 20:80, at the concentration of 2.5 mg/mL), DEX (100 μM), and combinations of cHA with DEX (100 μM) in response to IL-1β (1 ng/mL) stimulation. Relative expressions of (**A**) IL-1β (**B**) IL-6 (**C**) cyclooxygenase-2 (COX-2) (**D**) matrix proteinase-1 (MMP)-1, and (**E**) MMP-3 were determined by quantitative reverse transcription polymerase chain reaction (qRT-PCR). Values are the mean ± SEM (*n* = 2–3). * *p* < 0.05, ** *p* < 0.01, *** *p* < 0.001.

We next examined the inflammatory cytokine and MMP expressions in tenocytes under the three cHA + DEX formulations in response to IL-1β stimulation. After 24-h treatment, IL-1β surged the expression levels of IL-1β, IL-6, COX-2, MMP-1 and MMP-3 in human primary tenocytes, and their levels were significantly reversed after being treated with DEX. In the treatment of the three cHA + DEX formulations, down-regulation of the markers could be significantly maintained in the cHA (50:50)+ DEX and cHA (20:80) + DEX (*p* < 0.05, Figure 2).

### 2.3. Senescence and Apoptosis in Tenocytes

**Figure 3:**
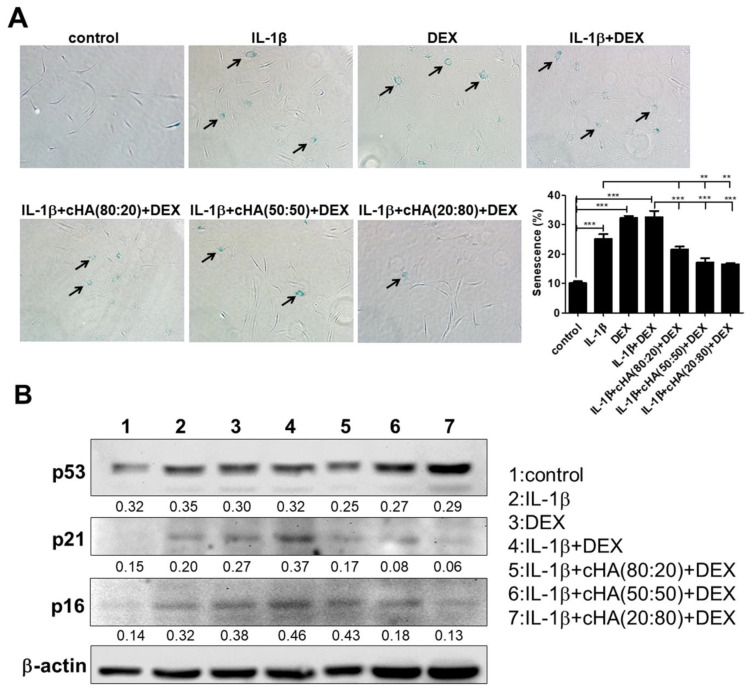
Effects of cHA with DEX in cellular senescence. Human primary tenocytes were treated with different formulations of cHA (cHA:linearized HA = 80:20, or 50:50, or 20:80, at the concentration of 2.5 mg/mL), DEX (100 μM), and combinations of cHA with DEX (100 μM) in response to IL-1β (1 ng/mL) stimulation. (**A**) Senescence β-galactosidase (SA β-gal) staining showed positive cells in IL-1β, DEX-, combinations of cHA with DEX-treated tenocytes. Arrows indicate SA β-gal-positive cells. (**B**) Immunoblotting for p53, p21, and p16 expressions in the three formulations of cHA with DEX. Values are the mean ± SEM (*n* = 3). ** *p* < 0.01, *** *p* < 0.001.

**Figure 4:**
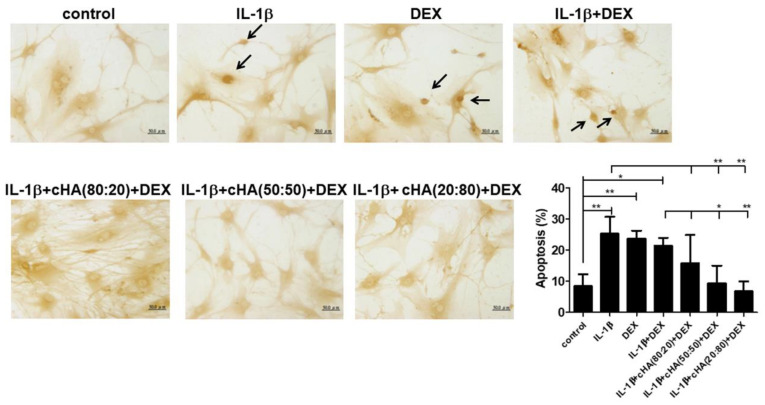
Effect of cHA and DEX in apoptosis. Human primary tenocytes were treated with different formulations of cHA (cHA:linearized HA = 80:20, or 50:50, or 20:80, at the concentration of 2.5 mg/mL), DEX (100 μM), and combinations of cHA with DEX (100 μM) in response to IL-1β (1 ng/mL) stimulation. Terminal deoxynucleotidyl transferase dUTP nick end labeling (TUNEL) staining showed positive cells in IL-1β, DEX-, combinations of cHA with DEX-treated tenocytes. Arrows indicate TUNEL-positive cells. Values are the mean ± SEM (*n* = 3–5). * *p* < 0.05, ** *p* < 0.01.

According to the data above, reduced cell viability was observed in human primary tenocytes upon treatment with DEX and IL-1β (Figure 1A). To further clarify the possible mechanisms, the senescent and apoptotic statuses were determined after a 5-day tenocyte culture. The ratios of senescence- and apoptosis-positive primary human tenocytes were significantly increased in IL-1β, DEX, and IL-1β + DEX- treated groups. In the treatments of cHA (50:50) + DEX and cHA (20:80) + DEX, both ratios could be significantly decreased compared with either IL-1β or IL-1β + DEX-treated group (Figure 3A and Figure 4). The expressions of the senescence markers, p53, p21, and p16, were increased in the IL-1β, DEX, and IL-1β+ DEX treated tenocytes, and higher following the IL-1β + DEX treatment. Similarly, in the treatments of cHA (50:50) + DEX and cHA (20:80) + DEX, all the expressions of the senescence markers were decreased (Figure 3B).

### 2.4. In Vivo Therapeutic Effects on a Rat Tendinopathy Model

**Figure 5:**
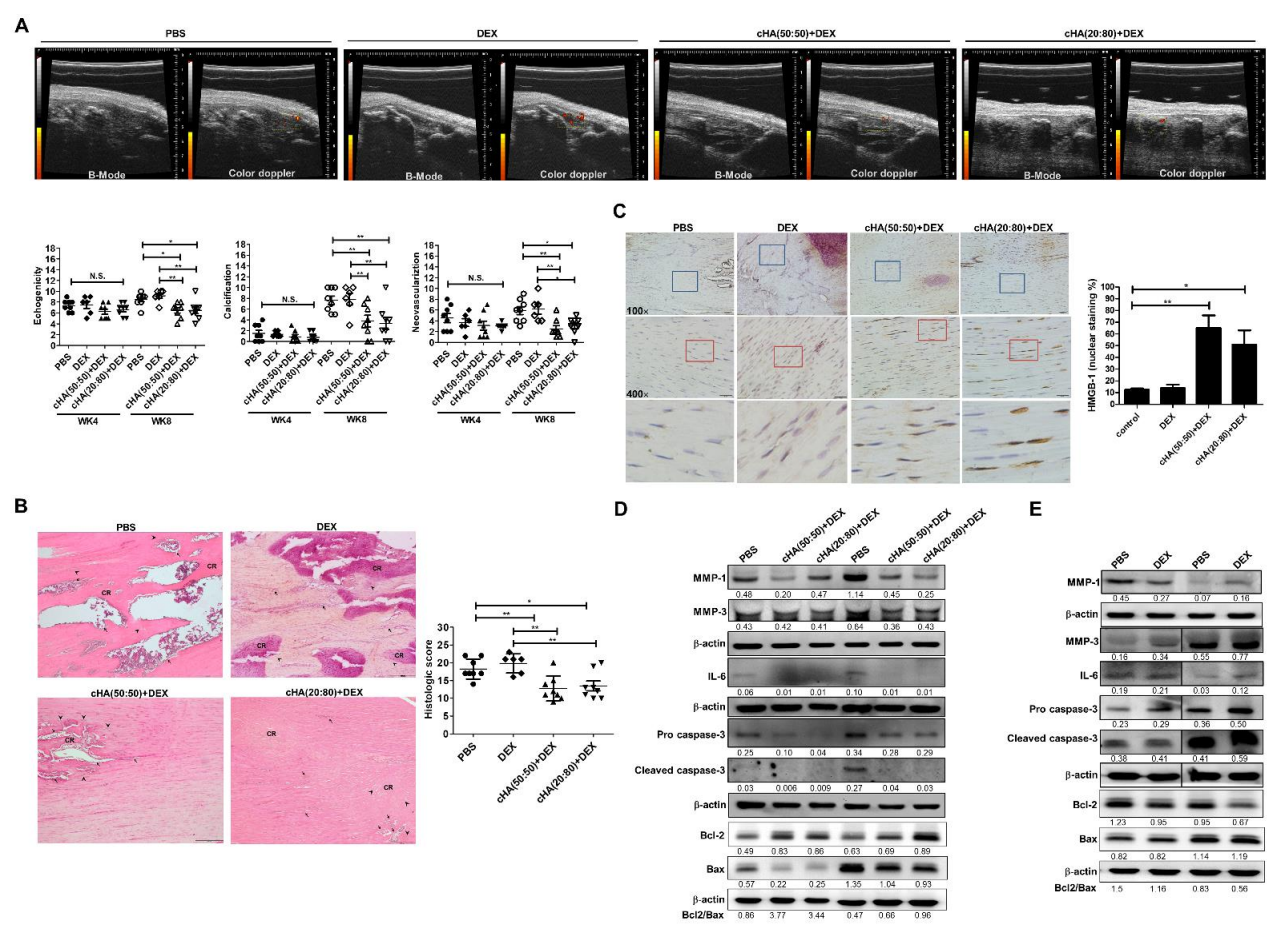
In vivo therapeutic effects and gene expressions in the experimental rat model. Four weeks after the intratendinous collagenase injection, the successfully induced rats under ultrasound examination were randomly allocated into four groups: PBS (control, *n* = 8), DEX only (*n* = 6), cHA (50:50) + DEX (*n* = 8), and cHA (80:20) + DEX (*n* = 8). Para-tendinous injection was conducted according to the allocation. (**A**) Ultrasound examination was performed 4 and 8 weeks after collagenase injection. The B-mode and color doppler images at week 8 were demonstrated. The semiquantitative scores of ultrasound features were measured and recorded. (**B**) Hematoxylin and eosin (H&E) stainings and histologic scores of control (PBS)-, DEX-, cHA (50:50) + DEX-, and cHA (80:20) + DEX-treated tendons. Bars shown at ×100 magnification correspond to 200 μM. CR: calcified region; arrowhead: chondrocyte-like cell; arrow: neovascularization. (**C**) Immunohistochemical staining of high mobility group box 1 (HMGB1) in the patellar tendons treated with PBS, DEX, cHA (50:50) + DEX, and cHA (20:80) + DEX. Blue boxed areas were shown at higher magnification in the panels (×400) beneath them. Red boxed areas were shown at higher magnification in the panels beneath them. Bars shown at ×100 and ×400 magnifications correspond to 50 and 20 μM. Expression levels of IL-6, pro caspase-3, cleaved caspase-3, MMP-1&-3 treated with (**D**) PBS, cHA (50:50) + DEX, and cHA (20:80) + DEX or in the patellar tendons (*n* = 2 in each group) treated with (**E**) PBS and DEX, as determined by immunoblotting. Values are the mean ± SEM. * *p* < 0.05, ** *p* < 0.01.

**Figure 6:**
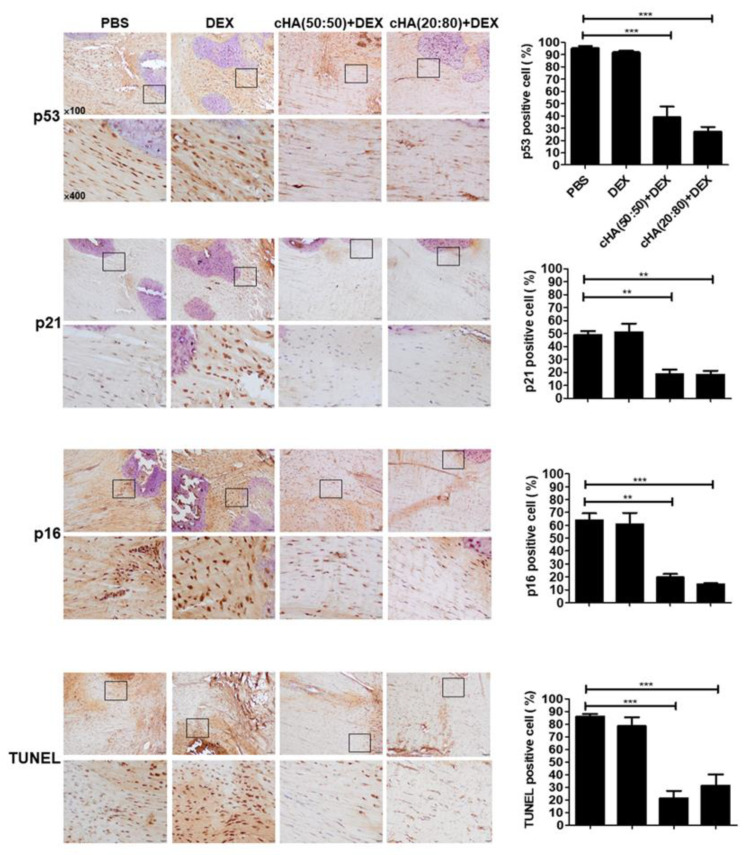
Immunohistochemistry of senescence-related markers and TUNEL analysis in the experimental rat model. Eight weeks after the intratendinous collagenase injection, the rats were scarified for further analysis. Immunohistochemical stainings and quantitative analysis of p53, p21, p16, and TUNEL were performed in the patellar tendons treated with PBS, DEX, cHA (50:50) + DEX, and cHA (20:80) + DEX. Bars shown at ×100 and ×400 magnifications correspond to 50 and 20 μM. Values are the mean ± SEM. ** *p* < 0.01, *** *p* < 0.001.

According to the in vitro data, cHA (50:50) + DEX and cHA (20:80) + DEX were chosen to evaluate the in vivo effects. Four weeks after collagenase injection, an ultrasound (US) examination was conducted before treatment and revealed that there was no significant difference in either US parameters among groups (Figure 5B). Following according treatments, both cHA (50:50) + DEX and cHA (20:80) + DEX groups (*n* = 8 in each group) revealed significantly lower US feature scores, including echogenicity, neovascularization, and calcification, than the PBS (control, *n* = 8) and DEX groups (*n* = 6) (all *p* < 0.05, Figure 5A,B). In the histological scores, the two cHA + DEX-treated tendons had significantly lower scores than the PBS and DEX-treated counterparts (*p* < 0.05; Figure 5B). High mobility group box 1 (HMGB1) belongs to the alarmin family and functions intracellularly, but upon cellular stress or damage are actively secreted by senescent cells, which happens very early after a senescence-inducing stimulus, before the development of the SASP [27,28]. Immunohistochemistry showed a significantly higher nuclear staining ratio of HMGB1 in the two cHA + DEX-treated tendons compared with the PBS and DEX-treated tendons (*p* < 0.05, Figure 5C). The in vivo protein levels of IL-6, cleaved caspase-3, pro caspase-3, MMP-1, and MMP-3 were lower in the two cHA + DEX groups than the PBS-treated groups (Figure 5D). However, their levels remained fairly or increasingly expressed in the DEX-treated tendinopathic tendons compared with the PBS groups, as determined by immunoblotting (Figure 5E). The protein ratios of Bcl2 by Bax were higher in the two cHA + DEX groups than those in the PBS-treated groups (Figure 5D), whereas the ratios were lower in the DEX-treated tendinopathic tendons compared with the PBS group (Figure 5E). The positive cell ratios of p53, p21, p16 and TUNEL were significantly lower in the two cHA + DEX groups than the PBS and DEX-treated groups, as determined by immunohistochemistry and TUNEL analyses (*p* < 0.01, Figure 6).

## 3. Discussion

In this study, we used three specific formulations of cHA + DEX to treat IL-1β-stimulated primary human tenocytes and the experimental rat tendinopathy model to test our hypothesis. The expression levels of inflammatory mediators, including IL-1β, IL-6, and COX-2, MMP-1, and MMP-3 were significantly reduced in all cHA + DEX-treated tenocytes. The two combinations, cHA (50:50) + DEX and cHA (20:80) + DEX, were identified to confer the protective effects from DEX-induced cytotoxicity, cellular senescence, and apoptosis in human tenocytes. Furthermore, the two combinations significantly improved the experimental tendinopathy by reducing ultrasound feature scores and histological scores as well as the levels of apoptosis, senescence, and senescence-associated secretory phenotypes. Both cHA (50:50) + DEX and cHA (20:80) + DEX ameliorate rat tendinopathy via anti-apoptotic and senolytic effects without obvious cytotoxicity.

Corticosteroid had shown its benefits for tendinopathy in the short term [11] and provides significant short-term pain relief and functional improvement for rotator cuff tendinopathy in a meta-analysis [12]. In clinical practice, the fast therapeutic response increases the frequency in combination with other injectable therapeutics, such as HA or plasma-rich platelet (PRP). The faster pain relief was reported by the combination of HA and corticosteroid for knee OA [23,24,25], adhesive capsulitis [26], and periarticular shoulder disorder [29]. However, the possible cytotoxicity induced by corticosteroids should be a concern. Wong et al. [30] demonstrated that DEX at 10^−3^ to 10^−9^ M decreased cell viability of tenocytes from normal patellar tendons in a dose-dependent fashion. In our results, the decreased tenocyte viability induced by DEX (100 μM) was significantly reversed by all the combinations of cHA + DEX, which was similar to the findings by Spitzer et al. [31] that the DEX (0.9~9000 μM)-loaded HA did not evoke cell death in human tenon fibroblasts. On the other hand, the increased synthesis of type III collagen is regarded as a repair response that would lead to contracture formation [32], adhesion [33], and even increased risk of tendon rupture [34]. A higher ratio of COLI/COLIII is considered to occur in the later remodeling phase [35]. In our results, the increased COL3A1 expression induced by IL-1β could be decreased following all the three cHA + DEX formulations. Finally, the ratio of COL1A1/COL3A1 was significantly improved in the cHA (50:50) + DEX-treated tenocytes compared with the IL-1β and IL-1β + DEX-treated ones. Our results indicated the combination of cHA + DEX could protect human primary tenocytes from the possible cytotoxicity by DEX and decrease type III collagen production.

Excessive apoptosis has been reported as a primary cause of tendinopathy [36] and found in tendinopathic rotator cuff [36,37], patellar tendons [38], and Achilles tendons [39]. Apoptotic phenotype is also positively correlated with the severity of tendinopathy [4,40]. The modulation of apoptosis via various approaches has shown therapeutic potential in experimental tendinopathy [7,41]. In our results, excessive apoptosis following IL-1β, DEX, and IL-1β + DEX inductions in primary human tenocytes can be reversed by cHA (50:50) + DEX and cHA (20:80) + DEX (Figure 4). In the experimental rat tendinopathy model, the ratios of TUNEL-positive cells in both the cHA + DEX groups were significantly decreased compared with PBS or DEX groups (Figure 6). Furthermore, in both the cHA + DEX groups, the ratios of Bcl-2/Bax were higher and correspondingly the levels of cleaved caspase-3 were lower (Figure 5D). Therefore, the reduction in excessive apoptosis might be one of the therapeutic mechanisms by the cHA + DEX treatment. Accordingly, the therapeutic effects improved the US features including hypoechogenicity and neovascularization, the two common parameters in clinical US assessment for tendinopathy [42].

Cellular senescence is the hallmark of aging and has been emerging for dissecting the pathogenic mechanisms of musculoskeletal disorders. Increased expression of proinflammatory mediators, such as IL-6 [43], and matrix-degrading enzymes, including MMP-1 and -3 [44], are associated with the senescence-associated secretory phenotype (SASP), which is an important mechanism in OA [45,46,47]. Jeon et al. reported that selective removal of senescent chondrocytes attenuated the development of post-traumatic OA, reduced pain and decreased expression of inflammatory markers as well as increased expression of cartilage tissue extracellular matrix proteins [27]. Our recent study reveals that senescence is positively correlated with disease severity of tendinopathy [6]. Decreased senescence and SASP via overexpression of CD44 can ameliorate rat experimental tendinopathy [6]. In this study, senescent markers and SASP induced by IL-1β, DEX, and IL-1β + DEX in human tenocytes can be reduced by the cHA (50:50) + DEX and cHA (20:80) + DEX treatments (Figure 3). In the rat tendinopathy model, the two specific cHA + DEX formulations significantly increased the ratio of cells with HMGB-1 nuclear staining (Figure 5C), and decreased the ratios of p53-, p21-, and p16-positive cells (Figure 6). Furthermore, the levels of SASP markers including IL-6, MMP-1, and MMP-3 were reduced following cHA (50:50) + DEX and cHA (20:80) + DEX treatments. Senolysis has been showed to ameliorate various age-associated disorders [48]. Integration of our previous study [6] and the current work, senolysis of tenocyte can improve tendinopathic characteristics. Taken together, we proposed that the specific cHA + DEX combination might be an ideal therapeutic agent for tendinopathy via the senolytic effect.

There are limitations of this study. First, even the in vitro and in vivo data support the therapeutic effects of cHA (50:50) + DEX and cHA (20:80) + DEX in tendinopathy, the downstream intracellular pathway of cHA + DEX responsible for modulation of apoptosis and senescence remained unclear. Furthermore, failed anti-senescent and -apoptotic effects by cHA (80:20) + DEX suggested the therapeutic potential of the specific cHA + DEX combination did not come from a simple physical property. Further explorations on detailed molecular mechanisms are needed to better understand the cHA + DEX treatment in tendinopathy. Second, the function-related parameters such as biomechanical properties and pain behavior were not evaluated in this study. The improvements in functional perspectives can confirm the therapeutic potential.

In conclusion, our results revealed the two formulations, cHA (50:50) + DEX and cHA (20:80) + DEX, ameliorate tendinopathy via anti-apoptotic and anti-senescent effects in IL-1β-stimulated tendinopathic tenocytes and a rat collagenase-induced tendinopathy model. This study provides a new approach to treat tendinopathy using the combination of two previously well-known agents. Further clinical studies are necessary to confirm the therapeutic effects of cHA and corticosteroid combination in patients with tendinopathy.

## 4. Materials and Methods

### 4.1. Ethic Statement

All the experimental rats were purchased from LASCO, Taiwan, and the following animal experiments were conducted strictly in accordance with protocols approved by the Institutional Animal Care and Use Committee of National Cheng Kung University (IACUC No. 106187). The human study was approved by the Institutional Review Board of National Cheng Kung University Hospital (IRB No.: A-ER-106-163) and was conducted strictly in accordance with the approved guidelines. Informed consent was obtained from all patients.

### 4.2. Preparation of cHA and cHA + DEX Formulations

Sodium hyaluronate (fermentation, purity > 95%, average molecular weight is about 1500 kDa) was cross-linked with 1,4-butanediol diglycidyl ether from Maxigen Biotech Inc. (Taoyuan, Taiwan). After cross-linking, the cHA was mixed with non-crosslinked HA by ratios of 80:20, 50:50, and 20:80 to prepare cHA (80:20), cHA (50:50) and cHA (20:80). Following the preparation of distinct cHA + DEX formulations, the DEX (Cat# SI-D2915, Sigma-Aldrich, St. Louis, MO, USA) was mixed with cHA (80:20), cHA (50:50) and cHA (20:80) by ratio of 1:4 (*v*/*v*) to prepare cHA (80:20) + DEX, cHA (50:50) + DEX and cHA (20:80) + DEX for the following cell viability, senescence and terminal deoxynucleotidyl transferase (TdT) dUTP nick end labeling (TUNEL) analyses. In all preparations, DEX was prepared at a final concentration of 100 μM.

### 4.3. Primary Culture of Human Tenocytes

Six consecutive patients (3 men, 3 women; median age: 63 years range: 52–69 years) undergoing arthroscopic treatment for a rotator cuff tear and long head of biceps (LHB) tendinopathy at our university hospital were recruited. The tenodesis or tenotomy of LHB were performed, and the pathological area of LHB was collected for the primary culture. The preparation of tendon samples and tenocyte culture methods were performed according to our previous study [6,49]. Well-characterized second-to-fourth passage cells were used in this experiment, and they showed no phenotypic drift of major tenocyte markers such as cell shape (elongated and spindle-shaped with apposition) and tenomodulin expression identified using anti-tenomodulin antibody (Santa Cruz) as described previously [6,49].

### 4.4. Cell Viability, Senescence and TUNEL Analyses

Primary human tenocytes were seeded into 96-well dishes and then treated with cHA (2.5 mg/mL), DEX (100 μM), or cHA + DEX or left untreated for 5 days. A WST-8 assay (Biovision/Abcam, Cambridge, UK) was used for evaluating cell viability in response to various treatments. Cell viability was represented as the percentages normalized with the average WST-8 values of the control group. Cells treated with IL-1β (1 ng/mL), DEX, IL-1β + DEX, and various formulations of cHA + DEX were subjected to β-galactosidase activity (Cell Signaling Technology, Danvers, MA, USA) and TUNEL (Promega, Madison, WI, USA) analyses for evaluating senescent and apoptotic statues in each group, according to the manufacturer’s instructions. Percentages of apoptosis and senescence were represented by counting the numbers of TUNEL-and β-galactosidase-positive cells relative to total cell numbers under the microscope (400× magnification).

### 4.5. Collagenase-Induced Patellar Tendinopathy Model

The tendinopathy rat model was modified from that described previously [50,51]. Male Sprague-Dawley rats (8 weeks old; weight, 250–300 g) were intra-tendinously injected with 10 μL (0.015 mg/μL in 0.9% saline) of bacterial collagenase I (Sigma-Aldrich, St. Louis, MO, USA) into their left patellar tendons under US-guidance. Four weeks after induction, all rats underwent the US examination for evaluation of tendinopathy, and the thickness of patellar tendons was recorded. If the increased thickness of the induced patellar tendon is less than 50% compared with the contralateral side, the rat was regarded as a failure of induction and excluded from the following treatment and analysis. The successfully induced rats were randomly allocated into four groups: phosphate-buffered saline (PBS), DEX, and two well-performing formulations of cHA + DEX according to the in vitro results. For injection treatment, the needle was aimed at the inferior paratendon area of the patellar tendon for prevention of intratendinous injection under the US guidance. Eight weeks after collagenase injection, rats were examined with US again and then sacrificed for further analyses (Supplementary Figure S1). Using the US (Vevo 770; VisualSonics, Toronto, ON, Canada) 55-MHz linear transducer for high-resolution images, the three common tendinopathic ultrasound features, including echogenicity, neovascularization, and calcification under real-time B-mode and color doppler were scored from 0 to 10 as previously described [50,51].

### 4.6. Histopathological Analysis

The rat tendons were fixed in fresh 4% paraformaldehyde for 16–24 h at 4 °C, and then subsequently dehydrated, paraffin-embedded, and longitudinally sectioned. Sequential 4-μM sections were stained with hematoxylin and eosin (H&E) and examined under a light microscope for changes in tenocyte morphology and collagen bundle characteristics. The histopathological severity was assessed using a 4-point system on the following eight parameters: fiber structure, fiber arrangement, the roundness of the nuclei, regional variations in cellularity, increased vascularity, decreased collagen stainability, fibrosis or hyalinization, and calcification characteristics [50,51]. The maximum total score was 24. The histological score was assessed by two observers unaware of the experimental setting. If an inconsistency existed, the field was reassessed, and a final score was decided upon.

### 4.7. Immunoblot and Immunohistochemical Analyses

Cell lysates of human tenocytes and rat tendons receiving various treatments (medium or PBS, cHA + DEX, IL-1β, and DEX alone) were subjected to immunoblot analysis with antibodies against p53 (#sc-6243, Santa Cruz Biotechnology, Dallas, TX, USA), p21 (#sc-6246, Santa Cruz Biotechnology), p16 (#sc-1661, Santa Cruz Biotechnology), IL-6(#GTX110527, GeneTex, Irvine, CA, USA), caspase-3 (#9662, Cell Signaling Technology, Danvers, MA, USA), cleaved caspase-3 (#9664, Cell Signaling Technology), MMP-1 (#GTX00674, GeneTex), MMP-3 (#14351, Cell Signaling Technology), Bcl-2 (#sc-7382, Santa Cruz Biotechnology), and Bax (#sc-7480, Santa Cruz Biotechnology), in combination with horseradish peroxidase–conjugated secondary antibody (GeneTex) and quantitative control anti-β-actin antibodies (#A3854, Sigma-Aldrich, St. Louis, MO, USA). Protein–protein complexes were visualized with an ECL Plus System (Amersham, UK) and analyzed with a BioSpectrum Imaging System, UVP, for chemiluminescence detection. For immunohistochemical staining, the sections were deparaffinized in xylene, dehydrated in alcohol, epitope-unmasking by heating, immersed in H_2_O_2_, and stained with antibodies against HMGB-1 (#sc-135809, Santa Cruz Biotechnology), p53 (#2524s, Cell Signaling Technology), p21 (#sc-6246, Santa Cruz Biotechnology), and p16 (#sc-1661, Santa Cruz Biotechnology), in combination with the chromogen 3-amino-9-ethylcarbazole (Zymed Laboratories Inc./ThermoFisher Scientific, Waltham, MA, USA).

### 4.8. Quantitative Reverse Transcription Polymerase Chain Reaction (qRT-PCR)

Total RNA from tenocytes under various treatments was isolated with TRIzol reagents (Invitrogen/ThermoFisher Scientific, Waltham, MA, USA), and cDNA was synthesized by using Reverse-iT First-strand Synthesis kit (ABgene/ThermoFisher Scientific, Waltham, MA, USA) for qRT-PCR bySYBR^®^ Premix Ex Taq™ (Takara, Kusatsu, Japan) with primer pairs specific to collagen type I alpha1 (COL1A1) (forward 5′-GCTATGATGAGAAATCAACCG -3′ and reverse 5′-TCATCTCCATTCTTTCCAGG -3′), collagen type III alpha1 (COL3A1) (forward 5′-ATTCACCTACACAGTTCTGG-3′ and reverse 5′-TGCGTGTTCGATATTCAAAG-3′), MMP-1 (forward 5′-AAAGGGAATAAGTACTGGGC-3′ and reverse 5′-CAGTGTTTTCCTCAGAAAGAG-3′), MMP-3 (forward 5′-GCAGTTAG AGAACATGGAG-3′ and reverse 5′-ACGAGAAATAAATTGGTCCC-3′), IL-1β (forward 5′- CTAAACAGATGAAGTGCTCC-3′ and reverse 5′-GGTCATTCTCCTGGAAGG-3′), IL-6 (forward 5′-GCAGAAAAAGGCAAAGAATC-3′ and reverse 5′-CTACATTTGCCGAAGAGC-3′), COX-2 (forward 5′-AAGCAGGCTAATACTGATAGG-3′ and reverse 5′-TGTTGAAAAGTAGTTCTGGG-3′), and GAPDH (forward 5′-AACATCATCCCTGCCTCTACTG-3′ and reverse 5′- CTCCGACGCCTGCTTCAC-3′). The comparative Ct method was used to calculate the relative abundance of each gene compared with GAPDH expression.

### 4.9. Statistical Analysis

Data are expressed as mean ± SEM. Normality was passed in each data by the Shapiro–Wilk test. Differences among groups were analyzed using one-way ANOVA and Dunnett’s tests as the post hoc test (Prism5.0, GraphPad Software Inc., La Jolla, CA, USA). Significance was set at *p* < 0.05.

## Data Availability

The data that support the findings of this study are available on request from the corresponding author.

## Supplementary Materials

The following supporting information can be downloaded at: https://www.mdpi.com/article/10.3390/ijms23179760/s1.
