# Supplementary material for: Cross-Linked Hyaluronate and Corticosteroid Combination Ameliorate the Rat Experimental Tendinopathy through Anti-Senescent and -Apoptotic Effects
Source: Int J Mol Sci. 2022 Aug 28;23(17):9760. doi: 10.3390/ijms23179760 (PMC9456262; doi:10.3390/ijms23179760)
Supplement: Supplementary file 1 [file ijms-23-09760-s001.zip › ijms-1870071-supplementary.pdf]

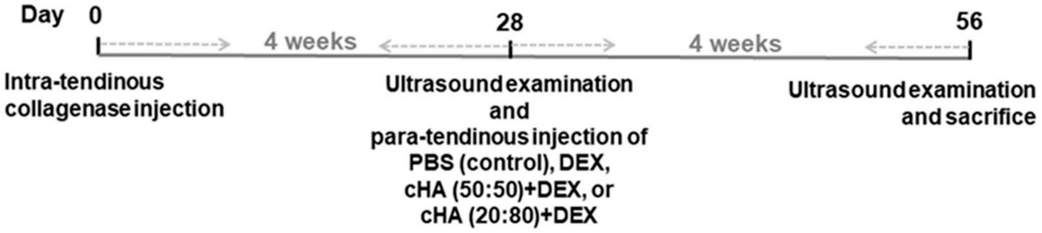

Supplementary Figure S1. A schematic representation of the procedures for animal study. Four weeks after collagenase injection into patellar tendons, the rats were examined with ultrasound and randomly divided into four groups receiving PBS (control), DEX, cHA(50:50)+DEX, or cHA(20:80)+DEX injection treatment. Eight weeks after collagenase injection, the rats received the 2nd ultrasound examination and then were sacrificed for further histologic examination and immunoblot analysis.
